# Bounding the *HL*-index of a graph: a majorization approach

**DOI:** 10.1186/s13660-016-1234-6

**Published:** 2016-11-17

**Authors:** Gian Paolo Clemente, Alessandra Cornaro

**Affiliations:** Department of Mathematics and Econometrics, Catholic University, Milan, Italy

**Keywords:** graph eigenvalue, median eigenvalue, majorization, HOMO-LUMO

## Abstract

In mathematical chemistry, the median eigenvalues of the adjacency matrix of a molecular graph are strictly related to orbital energies and molecular orbitals. In this regard, the difference between the occupied orbital of highest energy (HOMO) and the unoccupied orbital of lowest energy (LUMO) has been investigated (see Fowler and Pisansky in Acta Chim. Slov. 57:513-517, 2010). Motivated by the HOMO-LUMO separation problem, Jaklič *et al.* in (Ars Math. Contemp. 5:99-115, 2012) proposed the notion of *HL*-index that measures how large in absolute value are the median eigenvalues of the adjacency matrix. Several bounds for this index have been provided in the literature. The aim of the paper is to derive alternative inequalities to bound the *HL*-index. By applying majorization techniques and making use of some known relations, we derive new and sharper upper bounds for this index. Analytical and numerical results show the performance of these bounds on different classes of graphs.

## Introduction

The Hückel molecular orbital method (HMO) (see [3]) is a methodology for the determination of energies of molecular orbitals of *π*-electrons. It has been shown that *π*-electron energy levels are strictly related to graph eigenvalues. For this reason, graph spectral theory became a standard mathematical tool of HMO theory (see [4–6] and [7]). Among the various *π*-electron properties that can be directly expressed by means of graph eigenvalues, one of the most significant is the so-called HOMO-LUMO separation, based on the gap between the highest occupied molecular orbital (HOMO) and the lowest unoccupied molecular orbital (LUMO). For more details as regards the HOMO-LUMO separation issue we refer the reader to [8–10] and [11].

In some recent works, Fowler and Pisanski ([1] and [12]) introduced an index of a graph that is related to the HOMO-LUMO separation. By analogy with the spectral radius, these authors proposed the notion of the HOMO-LUMO radius which measures how large in absolute value may be the median eigenvalues of the adjacency matrix of a graph. At the same time, an analogous definition is given in [2] that introduces the *HL*-index of a graph. Several bounds for this index have been proposed for some classes of graphs in [1] and [13]. Recently in [14] the authors provided some inequalities on the *HL*-index through the energy index.

The contribution of this paper is along those lines: we derive, through a methodology based on majorization techniques (see [15–17] and [18]), new bounds on the median eigenvalues of the normalized Laplacian matrix. Consequently, given the relation between the normalized Laplacian matrix and the adjacency matrix eigenvalues, we provide some new bounds for the *HL*-index. In particular, we employ a theoretical methodology proposed by Bianchi and Torriero in [19] based on nonlinear global optimization problems solved through majorization techniques. These bounds can also be quantified by using the numerical approaches developed in [20] and [21] and extended for the normalized Laplacian matrix in [22] and in [23].

Furthermore, another approach to derive new bounds makes use of the relation between *HL*-index and energy index. In particular, we take advantage of an existing bound on the energy index (see [24]) depending on additional information on the first eigenvalue of the adjacency matrix. This additional information is obtained here by using majorization techniques in order to provide new inequalities for the *HL*-index of bipartite and non-bipartite graphs.

The paper is organized as follows. In Section 2 some notations and preliminaries are given. Section 3 concerns the identification of new bounds for the *HL*-index. In particular, in Section 3.1, by the fact that the eigenvalues of normalized Laplacian matrix and adjacency matrix are related, we localize the eigenvalues of the normalized Laplacian matrix via majorization techniques. In Section 3.2 we find a tighter alternative upper bound for the *HL*-index by using the relation with energy index provided in [14] and an existing bound on energy index proposed in [24]. Section 4 shows how the bound determined in Section 3.2 improves those presented in the literature.

## Notations and preliminaries

In this section we first recall some basic notions on graph theory (for more details refer to [25]) and on the *HL*-index. Considering a simple, connected and undirected graph $G=(V,E)$ where $V=\{1, 2, \ldots, n\}$ is the set of vertices and $E\subseteq V\times V$ the set of edges, ${|E|=m}$. The degree sequence of *G* is denoted by $\pi=(d_{1},d_{2},\ldots,d_{n})$ and it is arranged in non-increasing order $d_{1}\geq d_{2}\geq \cdots \geq d_{n}$, where $d_{i}$ is the degree of vertex *i*.

Let $A(G)$ be the adjacency matrix of *G* and $D(G)$ be the diagonal matrix of vertex degrees. The matrix $L(G)=D(G)-A(G)$ is called Laplacian matrix of *G*, while $\mathcal{L}(G)=D(G)^{-1/2}L(G)D(G)^{-1/2}$ is known as the normalized Laplacian. Let $\lambda _{1}\geq \lambda _{2}\geq \cdots\geq \lambda _{n}$, $\mu _{1}\geq \mu _{2}\geq \cdots\geq \mu_{n}$ and $\gamma _{1}\geq \gamma_{2}\geq \cdots\geq \gamma_{n}$ be the set of real eigenvalues of $A(G)$, $L(G)$, and $\mathcal{L}(G)$, respectively. The following properties of spectra of $A(G)$ and $\mathcal{L}(G)$ hold:

$$\begin{aligned} &\sum^{n}_{i=1}\lambda _{i}= \operatorname{tr} \bigl(A(G) \bigr)=0;\quad\quad \sum ^{n}_{i=1}\lambda _{i}^{2}= \operatorname{tr} \bigl(A^{2}(G) \bigr)=2m; \quad \lambda _{1}\geq \dfrac{2m}{n}; \\ &\sum^{n}_{i=1}\gamma_{i}= \operatorname{tr} \bigl(\mathcal{L}(G) \bigr)=n; \quad\quad \sum ^{n}_{i=1}\gamma_{i}^{2}= \operatorname{tr} \bigl(\mathcal{L}^{2}(G) \bigr)=n+2\sum _{(i,j)\in E}\frac{1}{d_{i}d_{j}}; \quad\gamma_{n}=0;\ \gamma_{1}\leq 2. \end{aligned}$$

The eigenvalues involved in the HOMO-LUMO separation are $\lambda_{H}$ and $\lambda_{L}$, where $H= \lfloor \frac{n+1}{2} \rfloor$ and $L= \lceil \frac{n+1}{2} \rceil$.

The *HL*-index of a graph is defined in [2] as

$$ R(G)=\max \bigl(|\lambda_{H}|,|\lambda_{L}| \bigr). $$

In the following, we list some well-known results on this index. In [2] the authors show that, for every connected graph, $R(G)$ is bounded as

1 $$ 0 \leq R(G) \leq d_{1}. $$

Other bounds have been found for special classes of graphs in [1, 13] and [26]. Finally, [14] shows that for a simple connected graph

2 $$ 0 \leq R(G) \leq \frac{E(G)}{n}, $$

where $E(G)=\sum_{i=1}^{n}\vert \lambda _{i}\vert $ is the energy index of graph introduced by Gutman in [27].

By (2), the following bounds depending on *n* and *m* have been derived in [14] for non-bipartite and bipartite graphs, respectively:

3 $$\begin{aligned} &{0 \leq R(G) \leq \frac{2m}{n^{2}}+\frac{1}{n}\sqrt{(n-1) \biggl(2m- \frac{4m^{2}}{n^{2}} \biggr)},} \end{aligned}$$

4 $$\begin{aligned} &{0 \leq R(G) \leq \frac{4m}{n^{2}}+\frac{1}{n}\sqrt{(n-2) \biggl(2m- \frac{8m^{2}}{n^{2}} \biggr)}.} \end{aligned}$$

Other bounds have been proposed in [14] for non-bipartite and bipartite graphs depending only on *n*:

5 $$ 0 \leq R(G) \leq \frac{\sqrt{n}+1}{2} $$

and

6 $$ 0 \leq R(G) \leq \frac{\sqrt{n}+\sqrt{2}}{\sqrt{8}}. $$

We now recall the following results regarding nonlinear global optimization problems solved through majorization techniques. We refer the reader to [19] for more details as regards majorization techniques and for the proofs of Lemma 1 and Theorems 1 and 2 recalled in the following.

Let *g* be a continuous function, homogeneous of degree *p*, real, and strictly Schur-convex (see [28] for the definition of Schur-convex functions and related properties). Let us assume

$$S=\Sigma \cap \Biggl\{ \boldsymbol{x} \in \mathbb{R}_{+}^{N}: g(\boldsymbol{x})=\sum_{i=1}^{N}x_{i}^{p}=b \Biggr\} , $$

where *p* is an integer greater than 1, $b \in \mathbb{R}$, and

$$\Sigma=\Biggl\{ \boldsymbol{x} \in \mathbb{R}_{+}^{N}: x_{1} \geq x_{2} \geq \cdots \geq x_{N}, \sum _{i=1}^{N}x_{i}=a\Biggr\} . $$

The following fundamental lemma holds.

### Lemma 1

*Fix* $b \in \mathbb{R}$ *and consider the set* *S*. *Then either* $b=\frac{a^{p}}{N^{p-1}}$ *or there exists a unique integer* $1 \leq h^{*} < N$ *such that*

$$ \frac{a^{p}}{ ( h^{\ast }+1 ) ^{p-1}}< b\leq \frac{a^{p}}{ (h^{\ast } ) ^{p-1}}, $$

*where* $h^{\ast}= \lfloor \sqrt[p-1]{\frac{a^{p}}{b}} \rfloor $.

We can now deduce upper and lower bounds for $x_{h}$ (with $h=1,\ldots,N$) by solving the following optimization problems $P(h)$ and $P^{*}(h)$: 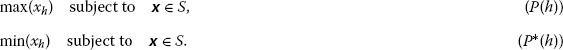


### Theorem 1

*The solution of the optimization problem* $P(h)$ *is* $(\frac{a}{N})$ *if* $b=\frac{a^{p}}{N^{p-1}}$. *If* $b \neq \frac{a^{p}}{N^{p-1}}$, *the solution of the optimization problem* $P(h)$ *is* $\alpha^{*}$ *where*

1. *for* $h>h^{*}$, $\alpha^{*}$ *is the unique root of the equation*
  7 $$ f(\alpha ,p)=(h-1)\alpha^{p}+(a-h\alpha +\alpha)^{p}-b=0 $$
  *in* $I=(0,\frac{a}{h} ]$;
2. *for* $h\leq h^{*}$, $\alpha^{*}$ *is the unique root of the equation*
  8 $$ f(\alpha,p)=h\alpha^{p}+\frac{(a-h\alpha)^{p}}{(N-h)^{p-1}}-b=0 $$
  *in* $I=( \frac{a}{N},\frac{a}{h} ]$.

### Theorem 2

*The solution of the optimization problem* $P^{*}(h)$ *is* $(\frac{a}{N})$ *if* $b=\frac{a^{p}}{N^{p-1}}$. *If* $b \neq \frac{a^{p}}{N^{p-1}}$, *the solution of the optimization problem* $P^{*}(h)$ *is* $\alpha^{*}$ *where*

1. *for* $h=1$, $\alpha^{*}$ *is the unique root of the equation*
  9 $$ f(\alpha,p) = h^{\ast }\alpha^{p}+ \bigl(a-h^{\ast }\alpha \bigr)^{p}- b= 0 $$
  *in* $I= ( \frac{a}{h^{\ast }+1},\frac{a}{h^{\ast }} ]$;
2. *for* $1 < h \leq (h^{*}+1)$, $\alpha^{*}$ *is the unique root of the equation*
  10 $$ f(\alpha,p) = (N-h+1)\alpha^{p}+ \frac{(a-(N-h+1)\alpha )^{p} }{(h-1)^{p-1}} - b=0 $$
  *in* $I=(0,\frac{a}{N}]$;
3. *for* $h >(h^{*}+1)$, $\alpha^{*}$ *is zero*.

## Some new bounds for the *HL*-index via majorization techniques

### Bounds on median eigenvalues of the normalized Laplacian matrix

In this section we localize median eigenvalues of the normalized Laplacian matrix by applying the methodology (see [19]) recalled in Section 2 (Theorems 1 and 2). The additional information on median eigenvalues and the interlacing between eigenvalues of normalized Laplacian and adjacency matrices turned out to be a handy tool for bounding the *HL*-index for both non-bipartite and bipartite graphs. According to [29] (see Theorem 2.2.1), the following relations hold:

11 $$ \frac{|\lambda_{n-k+1}|}{d_{1}} \leq |1-\gamma_{k}|\leq\frac{|\lambda_{n-k+1}|}{d_{n}}. $$

#### Proposition 1

*For a simple*, *connected*, *and non*-*bipartite graphs*

12 $$ 0 \leq R(G) \leq d_{1}\max\bigl(|1-\alpha_{1}|,|1- \beta_{1}|,|1-\alpha_{2}|,|1-\beta_{2}| \bigr) $$

*when* *n* *is even with*

$$\begin{aligned} &\alpha_{1}=\frac{1}{n-1} \biggl(n+\sqrt{\frac{n-2}{n} \bigl(b_{1}(n-1)-n^{2} \bigr)} \biggr),\\ & \beta_{1}= \frac{1}{n-1} \biggl(n-\sqrt{\frac{n-2}{n} \bigl(b_{1}(n-1)-n^{2} \bigr)} \biggr),\\ &\alpha_{2}=\frac{1}{n-1} \biggl(n+\sqrt{\frac{(n-4)}{(n+2)} \bigl(b_{1}(n-1)-n^{2}\bigr)} \biggr),\\ & \beta_{2}= \frac{n}{n-1} \biggl(1-\sqrt{\frac{b_{1}(n-1)-n^{2}}{n(n-2)}} \biggr), \end{aligned}$$

*and*

13 $$ 0 \leq R(G) \leq d_{1}\max \bigl(|1-\alpha_{3}|,|1- \beta_{3}| \bigr) $$

*when* *n* *is odd with*

$$ \alpha_{3}= \frac{1}{n-1} \biggl(n+\sqrt{\frac{(n-3)}{(n+1)} \bigl(b_{1}(n-1)-n^{2}\bigr)} \biggr), \qquad\beta_{3}= \frac{1}{n-1} \bigl(n-\sqrt{b_{1}(n-1)-n^{2}} \bigr). $$

#### Proof

From (11), we can easily derive the following bounds:

14 $$ 0 \leq R(G) \leq \left \{ \textstyle\begin{array}{l@{\quad}l} d_{1}\max (|1-\gamma_{\frac{n+2}{2}}|,|1-\gamma_{\frac{n}{2}}| ) & \text{ if $n$ is even,} \\ d_{1} (|1-\gamma_{\frac{n+1}{2}}| ) & \text{ if $n$ is odd.} \end{array}\displaystyle \right . $$

By applying majorization, we are able to bound the median eigenvalues $\gamma_{i}$ (with $i=\frac{n}{2},\frac{n+1}{2},\frac{n+2}{2}$) considered in (14).

To this aim, we face the set:

$$ S_{b}^{1}= \Biggl\{ \boldsymbol{\gamma} \in \mathbb{R}^{n-1}:\sum_{i=1}^{n-1} \gamma_{i}=n,g(\boldsymbol{\gamma})=\sum_{i=1}^{n-1} \gamma_{i}^{2}=b_{1}=n+2\sum _{(i,j)\in E}\frac{1}{d_{i}d_{j}} \Biggr\} . $$

It is well known that, for every connected graph of order *n* (see [30]), we have

$$ \frac{1}{n-1} \leq \frac{2}{n} \sum_{(i,j)\in E} \frac{1}{d_{i}d_{j}} < 1 $$

and, consequently,

15 $$ \frac{n^{2}}{n-1} \leq b_{1} < 2n, $$

where the left inequality is attained for the complete graph $G=K_{n}$.

It is noteworthy to see that $S_{b}^{1}$ is derived from the general set *S* with $a=n$, $N=n-1$, $b=b_{1}$, and^1^ $p=2$. By Lemma 1 we have for a non-complete graph

$$h^{\ast}= \biggl\lfloor \frac{n^{2}}{b_{1}} \biggr\rfloor $$

with

16 $$ \biggl\lfloor \frac{n}{2} \biggr\rfloor < h^{\ast} < n-1. $$

We distinguish the following cases:

1. Considering $\gamma_{\frac{n}{2}}$ for *n* even, by (16) we have $h < h^{\ast}$. Hence, by solving equation (8) of Theorem 1, we can derive the unit root $\alpha_{1}$ such as $\gamma_{\frac{n}{2}}\leq \alpha_{1}$. Therefore
  $$ \alpha_{1}=\frac{1}{n-1} \biggl(n+\sqrt{\frac{n-2}{n} \bigl(b_{1}(n-1)-n^{2} \bigr)} \biggr). $$
  In virtue of (15), we have $\frac{n}{n-1} \leq \alpha_{1} < 2$ with the left inequality attained only for the complete graph $G=K_{n}$.
  In a similar way, we can evaluate the value $\beta_{1} \leq \gamma_{\frac{n}{2}} $, through the solution of equation (10) of Theorem 2 (where $h< h^{\ast}+1$), where
  $$ \beta_{1}=\frac{1}{n-1} \biggl(n-\sqrt{\frac{n-2}{n} \bigl(b_{1}(n-1)-n^{2} \bigr)} \biggr). $$
  From (15), we have $\frac{2}{n-1} < \beta_{1} \leq \frac{n}{n-1}$ with the right inequality attained only for the complete graph $G=K_{n}$.
  Having $\beta_{1} \leq \gamma_{\frac{n}{2}} \leq \alpha_{1}$, then $\vert 1-\gamma_{\frac{n}{2}}\vert \leq \max(|1-\alpha_{1}|, |1-\beta_{1}|)$.
2. Picking now $\gamma_{\frac{n+2}{2}}$, where $h \leq h^{\ast}$ by equation (8) of Theorem 1 we deduce
  $$ \alpha_{2}=\frac{1}{n-1} \biggl(n+\sqrt{\frac{(n-4)}{(n+2)} \bigl(b_{1}(n-1)-n^{2}\bigr)} \biggr). $$
  Taking into account the lower bound of $\beta_{2} \leq \gamma_{\frac{n+2}{2}}$, by (16) and for *n* even, we have $h < h^{\ast}+1$ and then
  $$ \beta_{2}=\frac{n}{n-1} \biggl(1-\sqrt{\frac{b_{1}(n-1)-n^{2}}{n(n-2)}} \biggr). $$
  In this case, we derive $\vert 1-\gamma_{\frac{n+2}{2}}\vert \leq \max(|1-\alpha_{2}|, |1-\beta_{2}|)$.
3. For a graph with *n* odd number of vertices, we need to study only $\gamma_{\frac{n+1}{2}}$ with $\beta_{3} \leq \gamma_{\frac{n+1}{2}} \leq \alpha_{3}$, and we have by Theorem 1 and Theorem 2, respectively:
  $$ \alpha_{3}= \frac{1}{n-1} \biggl(n+\sqrt{\frac{(n-3)}{(n+1)} \bigl(b_{1}(n-1)-n^{2}\bigr)} \biggr) $$
  and
  $$ \beta_{3}=\frac{1}{n-1} \bigl(n-\sqrt{b_{1}(n-1)-n^{2}} \bigr), $$
  where $h < h^{\ast}+1$.
  Hence, $\vert 1-\gamma_{\frac{n+1}{2}}\vert \leq \max(|1-\alpha_{3}|, |1-\beta_{3}|)$.

By using the bounds on the eigenvalues of the normalized Laplacian matrix computed above, bounds (12) and (13) follow. □

#### Proposition 2

*For a simple*, *connected and bipartite graphs with* *n* *even we have*

17 $$ 0 \leq R(G) \leq d_{1}\bigl|1-\beta^{\mathrm{bip}}_{1}\bigr|, $$

*where*

$$ \beta^{\mathrm{bip}}_{1}=1-\sqrt{\frac{b_{2}}{n-2}-1}. $$

#### Proof

When *G* is bipartite, the *HL*-index is defined as

18 $$ R(G)=\left \{\textstyle\begin{array}{l@{\quad}l} |\lambda_{\frac{n}{2}}|=|\lambda_{\frac{n+2}{2}}| & \text{ if $n$ is even,} \\ 0 & \text{ if $n$ is odd} . \end{array}\displaystyle \right . $$

In virtue of (11), we can derive the following bound when *n* is even:

19 $$ R(G) \leq d_{1}\vert 1-\gamma_{\frac{n}{2}}\vert . $$

By applying majorization techniques, we are able to bound $\gamma_{\frac{n}{2}}$ considered in (19).

To this aim, we now face the set

$$ S^{2}_{b}= \Biggl\{ \boldsymbol{\gamma} \in \mathbb{R}^{n-2}:\sum_{i=2}^{n-1} \gamma_{i}=n-2,g(\boldsymbol{\gamma})=\sum _{i=2}^{n-1}\gamma_{i}^{2}=b_{2}=n+2 \sum_{(i,j)\in E}\frac{1}{d_{i}d_{j}}-4 \Biggr\} . $$

By (15) and $b_{2}=b_{1}-4$, we have

20 $$ \frac{(n-2)^{2}}{n-1} \leq b_{2} < 2(n-2), $$

where the left inequality is attained for the complete graph $G=K_{n}$.

The set $S_{b}^{2}$ is derived from the general set *S* with $a=n-2$, $N=n-2$, $b=b_{2}$, and $p=2$. By Lemma 1 we have for a non-complete graph

$$h^{\ast}= \biggl\lfloor \frac{(n-2)^{2}}{b_{2}} \biggr\rfloor $$

with

21 $$ \frac{n}{2}-1 < h^{\ast} \leq n-1, $$

for *n* even.

We now consider $\gamma_{\frac{n}{2}}$:

1. By (21), we have $h \leq h^{\ast}$. In virtue of equation (8) of Theorem 1, we deduce the following upper bound:
  $$ \alpha^{\mathrm{bip}}_{1}=1+\sqrt{ \biggl(\frac{n-4}{n} \biggr) \biggl(\frac{b_{2}}{n-2}-1 \biggr)} . $$
2. In a similar way, we can evaluate the value $\beta^{\mathrm{bip}}_{1} \leq \gamma_{\frac{n}{2}} $, where $h < h^{\ast}+1$. Applying Theorem 2 entails
  $$ \beta^{\mathrm{bip}}_{1}=1-\sqrt{\frac{b_{2}}{n-2}-1}. $$

It is easy to show that $|1-\alpha^{\mathrm{bip}}_{1}| \leq |1-\beta^{\mathrm{bip}}_{1}|$. Hence bound (17) follows. □

### Bounds on $R(G)$ through the energy index

In the following we obtain bounds on the *HL*-index starting from (2). Our aim is to bound the energy index making use of additional information on the first eigenvalue of $A(G)$. In [24] the authors show that, if a tighter bound *k* on $\lambda_{1}$ such as $\lambda_{1} \geq k \geq \frac{2m}{n}$ is available, then the energy index for a non-bipartite graph is bounded as

$$ E(G)\leq k + \sqrt{(n-1) \bigl(2m-k^{2}\bigr)}, $$

while for a bipartite graph it is

$$ E(G)\leq 2k + \sqrt{(n-2) \bigl(2m-2k^{2}\bigr)}. $$

In order to find the value of *k*, we can introduce new variables $x_{i}=\lambda_{i}^{2}$, facing the set:

$$ S^{2}_{b}= \Biggl\{ \boldsymbol{x} \in \mathbb{R}_{+}^{n}: \sum_{i=1}^{n}x_{i}=2m \Biggr\} . $$

In virtue of (2), we are now able to derive the following bounds for non-bipartite and bipartite graphs, respectively.

#### Proposition 3

1. *For a simple*, *connected and non*-*bipartite graph* *G*
  22 $$ R(G)\leq \frac{k}{n} + \frac{1}{n}\sqrt{(n-1) \bigl(2m-k^{2} \bigr)}. $$
2. *For a simple*, *connected and bipartite graph* *G*
  23 $$ R(G)\leq \frac{2k}{n} + \frac{1}{n}\sqrt{(n-2) \bigl(2m-2k^{2} \bigr)}, $$
  *where*, *by means of Theorem* 2,
  $$k=\frac{1}{1+h^{\ast}} \biggl(n+\sqrt{\frac{2m(1+h^{\ast})-n^{2}}{h^{\ast}}} \biggr),\quad h^{\ast}= \biggl\lfloor \frac{n^{2}}{2m} \biggr\rfloor .$$

#### Remark 1

Bounds (22) and (23) are tighter than or equal to (3) and (4), respectively.

#### Proof of Proposition 3

1. *Non-bipartite graphs*
  We start by proving that the condition $(2m-k^{2})\geq0$ required in bound (22) is always satisfied for simple and connected graphs.
  We have $k \in ( \frac{2m}{n},\frac{1}{2} (n+\sqrt{4m-n^{2}} ) )$. Indeed, by the basic concepts of calculus it is easy to see that *k* increases when *m* increases and then $h^{\ast}$ tends to 1. Hence, *k* is limited from above by $\frac{1}{2} (n+\sqrt{4m-n^{2}} )$ and where $\frac{1}{2} (n+\sqrt{4m-n^{2}} )\leq \sqrt{2m}$ the required condition is satisfied.
  We now show how bound (22) improves bound (3) presented in [14].
  We need to prove that the following inequality holds:
  $$ \biggl( k-\frac{2m}{n} \biggr)\leq \biggl( \sqrt{ ( n-1 ) \biggl( 2m- \frac{4m^{2}}{n^{2}} \biggr) }-\sqrt{ ( n-1 ) \bigl( 2m-k^{2} \bigr) } \biggr). $$
  By simple algebraic rules we obtain
  24 $$\begin{aligned} &k^{2}n-4k\frac{m}{n}-4mn+4m+4 \frac{m^{2}}{n} \\ &\quad\leq-2\sqrt{ \bigl( 2mn-2m+k^{2}-k^{2}n \bigr) \biggl( 2mn-2m-4 \frac{m^{2}}{n}+4\frac{m^{2}}{n^{2} } \biggr) }. \end{aligned}$$
  The left-hand side term of (24) can be represented by
  $$ f(k)=k^{2}n-4k\frac{m}{n}-4mn+4m+4\frac{m^{2}}{n}. $$
  The function $f(k)$ is a convex parabola that assumes negative values in the range of *k* we are interested in. Indeed we have $f(\frac{2m}{n}) \leq 0$ and $f(\sqrt{2m}) \leq 0$.
  Both sides of (24) being negative we can apply some basic concepts of algebra, getting
  25 $$ k^{2}n^{2}+k ( 4mn-8m ) +8m-8mn+4m^{2}\geq 0 . $$
  The function $t(k)=k^{2}n^{2}+k ( 4mn-8m ) +8m-8mn+4m^{2}$ is again a convex parabola with vertex $( \frac{2m ( 2-n ) }{n^{2}},\frac{8m ( 2m-n^{2} ) ( n-1 )}{n^{2}} ) $. Both coordinates are less than zero (then $\frac{2m (2-n ) }{n^{2}} < \frac{2m}{n}$). Having $t(\frac{2m}{n})=8m(n-2m)(1-n)\geq 0$, inequality (25) is satisfied.
  Therefore, bound (22) performs better than or equal to bound (3).
  Furthermore, we see that both bounds perform equally when $h^{\ast}= \frac{n^{2}}{2m}$ (*i.e.* $\frac{n^{2}}{2m}$ is an integer). It is noteworthy that:
  - when *n* is odd, $\frac{n^{2}}{2m}$ is never an integer ($\lfloor \frac{n^{2}}{2m} \rfloor \neq \frac{n^{2}}{2m}$);
  - when *n* is even, $\frac{n^{2}}{2m}$ is an integer when $m=\frac{n^{2}}{2x}$ with $x|\frac{n^{2}}{2}$, $2 \leq x \leq \frac{n}{2}$ (where $x|\frac{n^{2}}{2}$ is shorthand for ‘*x* divides $\frac{n^{2}}{2}$’). In this case $k=\frac{2m}{n}$ and we derive bound (3).
2. *Bipartite graphs*
  As for non-bipartite graphs, we start by proving that the condition $(m-k^{2})\geq0$ required in bound (23) is always satisfied for simple and connected graphs. In this case $h^{\ast}$ tends to 2 for complete graphs (where $m \leq \frac{n^{2}}{4}$).
  By some basic algebraic concepts, we see that $(m-k^{2})\geq0$ entails:
  $$m^{2}\bigl(h^{4}+2h^{3}-3h^{2}-4h+4\bigr)+m\bigl(2n^{2}h-4n^{2}-2n^{2}h^{2}\bigr)+n^{4} \geq 0. $$
  The function $f(m)=m^{2}(h^{4}+2h^{3}-3h^{2}-4h+4)+m(2n^{2}h-4n^{2}-2n^{2}h^{2})+n^{4}$ is concave, decreasing, and non-negative on the interval $m \in (\frac{n}{2},\frac{n^{2}}{4} )$. Therefore, the required condition is satisfied.
  We now show how bound (23) improves bound (4) presented in [14].
  We need to prove that the following inequality holds:
  $$ 2 \biggl( k-\frac{2m}{n} \biggr)\leq \biggl( \sqrt{ ( n-2 ) \biggl( 2m- \frac{8m^{2}}{n^{2}} \biggr) }-\sqrt{ ( n-2 ) \bigl( 2m-2k^{2} \bigr) } \biggr). $$
  By simple algebra, we have
  26 $$\begin{aligned} &4mn-8km+4m^{2}-2mn^{2}+k^{2}n^{2} \\ &\quad\leq-n\sqrt{ \bigl(2mn-4m+4k^{2}-2k^{2}n \bigr) \biggl(2mn-4m-8 \frac{m^{2}}{n}+16\frac{m^{2}}{n^{2}} \biggr)}. \end{aligned}$$
  The left-hand side term of (26) can be represented by
  $$ f(k)=k^{2}n^{2}-8km+4mn+4m^{2}-2mn^{2}. $$
  The function $f(k)$ is a convex parabola that assumes negative values in the range of *k* we are interested in. Indeed we have $f(\frac{2m}{n}) \leq 0$ and $f(\sqrt{m}) \leq 0$.
  Both sides of (26) being negative, by some manipulations we obtain
  27 $$ k^{2}n^{2}+4km(n-4)+4m^{2}+16m-8mn\geq 0 . $$
  The function $t(k)=k^{2}n^{2}+4km(n-4)+4m^{2}+16m-8mn$ is again a convex parabola with vertex $( \frac{2m (4-n ) }{n^{2}},\frac{8m(4m-n^{2})(n-2)}{n^{2}} )$. Both coordinates are less than zero (then $\frac{4m ( 4-n ) }{2n^{2}} < \frac{2m}{n}$). Having $t(\frac{2m}{n})=\frac{8m}{n}(n+2m(n-2))\geq 0$, inequality (27) is satisfied.
  Hence bound (23) performs better than or equal to bound (4).

 □

## Numerical results

### Non-bipartite graphs

In this subsection we compare our bounds ((12), (13), and (22)) with those existing in the literature. We initially compute them for graphs generated by using the Erdös-Rényi (ER) model $G_{\mathrm{ER}}(n,q)$ (see [31–33] and [34]) where edges are included with probability *q* independent from every other edge. Graphs have been derived randomly by using the well-known package of R (see [35]) and by ensuring that the graph is connected. Table 1 compares alternative upper bounds of $R(G)$ evaluated for simulated $G_{\mathrm{ER}}(n,0.5)$ graphs with different number of vertices. It is noteworthy that bound (13) has the best performance for $n=5$, while bound (22) is the sharpest one in all other cases. However, the improvements are very slight with respect to bound (3) when large graphs are considered.

**Table 1:** **Upper bounds of** $\pmb{R(G)}$ **for graphs generated by** $\pmb{G_{\mathrm{ER}}(n,0.5)}$ **model**

***n***	***m***	***R*** **(** ***G*** **)**	**Bound (** **12** **)/(** **13** **)**	**Bound (** **22** **)**	**Bound (** **1** **)**	**Bound (** **3** **)**	**Bound (** **5** **)**
5	7	0.46	1.29	1.33	4	1.55	1.62
10	26	0.68	1.99	1.80	8	2.02	2.08
15	52	0.33	3.12	2.28	10	2.33	2.44
20	106	0.69	3.98	2.27	16	2.71	2.74
25	153	0.32	3.66	2.92	16	2.94	3.00
50	600	0.42	5.10	3.92	31	3.98	4.04
100	2,463	0.56	7.30	5.43	66	5.47	5.50
250	15,358	0.49	9.67	8.32	142	8.38	8.41
500	62,304	0.47	13.52	11.65	289	11.67	11.68
1,000	249,556	0.50	17.92	16.29	549	16.30	16.31

We now extend the analysis to the same class of graphs but with varying the probability *q*. Two alternative values ($q=0.1$ and $q=0.9$, respectively) have been tested in Tables 2 and 3. In both cases bound (22) appears always as the tightest one. In particular, we observe significant differences with respect to other bounds when the number of edges increases. It is noticeable in Table 2 how the improvement is very remarkable even for very large graphs.

**Table 2:** **Upper bounds of** $\pmb{R(G)}$ **for graphs generated by** $\pmb{G_{\mathrm{ER}}(n,0.9)}$ **model**

***n***	***m***	***R*** **(** ***G*** **)**	**Bound (** **12** **)/(** **13** **)**	**Bound (** **22** **)**	**Bound (** **1** **)**	**Bound (** **3** **)**	**Bound (** **5** **)**
5	9	1.00	1.41	1.17	4	1.62	1.62
10	43	1.00	1.61	1.15	9	1.90	2.08
15	102	1.00	1.57	1.13	14	2.00	2.44
20	176	1.00	2.19	1.22	19	2.30	2.74
25	284	1.00	2.09	1.19	24	2.32	3.00
50	1,154	1.00	2.72	1.24	49	2.79	4.04
100	4,669	0.94	3.39	1.31	98	3.41	5.50
250	29,507	0.96	4.63	1.42	246	4.57	8.41
500	118,482	0.95	6.00	1.57	488	5.91	11.68
1,000	474,713	0.96	7.96	1.79	967	7.88	16.31

**Table 3:** **Upper bounds of** $\pmb{R(G)}$ **for graphs generated by** $\pmb{G_{\mathrm{ER}}(n,0.1)}$ **model**

***n***	***m***	***R*** **(** ***G*** **)**	**Bound (** **12** **)/(** **13** **)**	**Bound (** **22** **)**	**Bound (** **1** **)**	**Bound (** **3** **)**	**Bound (** **5** **)**
5	4	0.00	1.26	1.25	2	1.25	1.62
10	11	0.33	3.09	1.46	5	1.46	2.08
15	17	0.00	3.51	1.49	6	1.49	2.44
20	25	0.12	3.07	1.57	5	1.57	2.74
25	40	0.00	3.18	1.76	6	1.76	3.00
50	112	0.04	5.07	2.09	11	2.09	4.04
100	501	0.09	5.23	3.08	17	3.09	5.50
250	3,082	0.14	7.01	4.80	36	4.80	8.41
500	12,541	0.10	9.50	6.80	70	6.81	11.68
1,000	49,890	0.14	12.09	9.57	126	9.57	16.31

**Figure 1:**
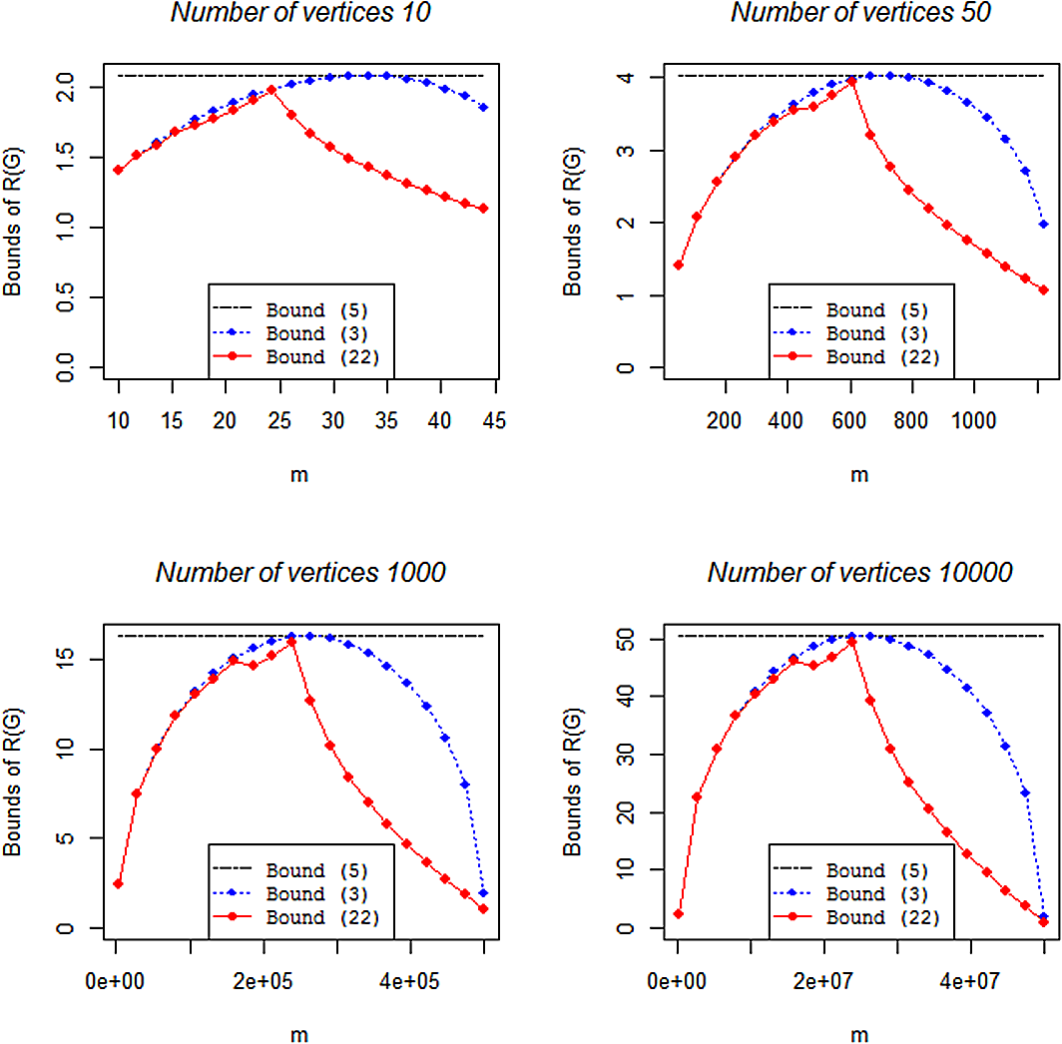
**Upper bounds of** $\pmb{R(G)}$ **for non-bipartite graphs with different number of vertices and edges.**

We now focus on bounds (3), (5), and (22) that share the advantage of being dependent only on *n* and/or *m*. In Section 3.2, it has been already proved that bound (22) is better than or equal to bound (3). This result is obviously confirmed by Figure 1, where it is interesting to highlight how bound (22) strongly improves the other bounds for large values of *m*.

### Bipartite graphs

We now report in Table 4 a comparison of alternative bounds derived for bipartite graphs, varying the number of vertices and edges. In some selected cases (*i.e.* $n=10$, $m=25$ and $n=20$, $m=91$), our bound (17) performs better. In all the other cases, the tightest one is bound (23) as obtained in Section 3.2.

**Table 4:** **Upper bounds of** $\pmb{R(G)}$ **for bipartite graphs**

***n***	***m***	***R*** **(** ***G*** **)**	**Bound (** **17** **)**	**Bound (** **23** **)**	**Bound (** **4** **)**	**Bound (** **6** **)**
10	13	0.46	1.75	1.47	1.52	1.62
10	25	0.00	0.00	1.00	1.00	1.62
20	45	0.10	2.97	1.90	1.94	2.08
20	91	0.00	1.04	1.16	1.77	2.08
50	315	0.06	3.82	2.86	2.95	3.00
50	560	0.00	1.74	1.34	2.39	3.00
100	1,248	0.03	4.72	4.00	4.01	4.04
100	2,254	0.03	2.36	1.48	2.99	4.04
250	7,839	0.02	7.52	5.91	6.07	6.09
250	14,083	0.03	3.60	1.79	4.22	6.09
500	31,321	0.04	9.29	8.20	8.39	8.41
500	56,231	0.01	5.01	2.16	5.64	8.41
1,000	124,887	0.02	12.68	11.66	11.67	11.68
1,000	225,145	0.01	7.02	2.65	7.58	11.68

**Figure 2:**
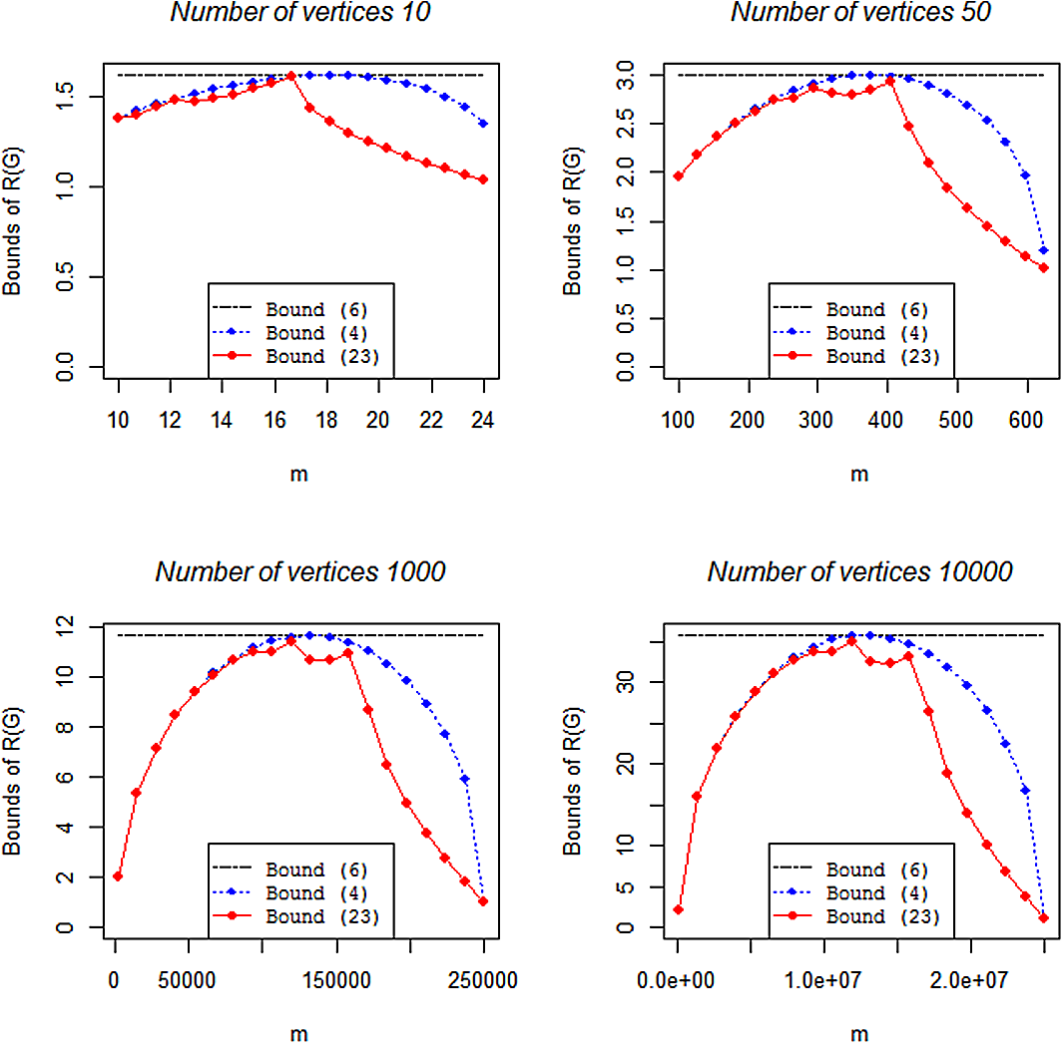
**Upper bounds of** $\pmb{R(G)}$ **for bipartite graphs with different number of vertices and edges.**

As for non-bipartite graphs, we can focus on bounds (4), (6), and (23) that can be computed without generating any graph. We observe in Figure 2 how the bound (23) provides a significant improvement for higher values of *m*. This result is also confirmed for very large graphs.

## Conclusions

In this paper alternative upper bounds on the *HL*-index are provided by means of majorization techniques. On one hand, we find new bounds for both non-bipartite and bipartite graphs by exploiting additional information on the median eigenvalues and the interlacing between the eigenvalues of normalized Laplacian and adjacency matrices. On the other hand, new bounds are derived by making use of the relation between the *HL*-index and the energy index. Analytical and numerical results show the performance of these bounds on different classes of graphs. In particular, the bound related to the energy index performs better with respect to the best-known results in the literature.

## Footnotes

1 For values of $p > 2$, *b* (and then $b_{1}$ and $b_{2}$ used in the sequel) depends on the graph’s structure and topology. So the procedure can be only numerically applied: we need to compute the eigenvalues of either adjacency or normalized Laplacian matrix, but this information allows one to directly obtain $R(G)$. In this case, the evaluation of the bounds is useless.
